# Sensorineural Hearing Loss Post-COVID-19 Infection: An Update

**DOI:** 10.3390/audiolres12030032

**Published:** 2022-06-01

**Authors:** Virginia Fancello, Giuseppe Fancello, Stavros Hatzopoulos, Chiara Bianchini, Francesco Stomeo, Stefano Pelucchi, Andrea Ciorba

**Affiliations:** 1 ENT & Audiology Unit, Department of Neurosciences, University Hospital of Ferrara, 44124 Ferrara, Italy; sdh1@unife.it (S.H.); chiara.bianchini@unife.it (C.B.); stmfnc@unife.it (F.S.); stefano.pelucchi@unife.it (S.P.); andrea.ciorba@unife.it (A.C.); 2 Department of Otorhinolaryngology, Careggi University Hospital, 50134 Florence, Italy; giuseppe.fancello@unifi.it

**Keywords:** COVID-19, sensorineural hearing loss, audiology

## Abstract

The course of COVID-19 infection may be complicated by a variety of neurological manifestations. Since the inner ear is vulnerable to viruses, sensorineural hearing loss (SNHL) has been reported to occur following the SARS-CoV-2 infection, often resulting in long-term morbidity and worsening the quality of life. The interest in how the virus affects the inner ear has gradually increased since the pandemic’s spread, but little is still known about the SNHL potentially caused by SARS-CoV-2. The aim of this paper is to evaluate the possible association between SNHL and COVID-19 infection, through a systematic literature review. Currently available data suggest that SARS-CoV-2 may hamper cochlear function; however, available reports are still limited. Large cohort and prospective studies are necessary to evaluate the long-term effects of this viral infection in the inner ear.

## 1. Introduction

The World Health Organization (WHO) declared the SARS-CoV-2 infection pandemic more than two years ago. Many people have been affected and many others are still suffering from the prolonged effects of SARS-CoV-2. In particular, neurological symptoms have been reported to be present in more than 80% of severe cases and could be related to the virus neurotropic and neuroinvasive properties [1].

Considering that the inner ear is reported to be vulnerable to viruses, it is not surprising that recent data in the literature link sensorineural hearing loss (SNHL), tinnitus, and/or vertigo, with SARS-CoV-2 infection. These symptoms might result in long-term morbidity and to a deterioration of the quality of life. The first link between SNHL and COVID-19 was proposed by Sriwijitalai [2] in April 2020, and subsequently, the focus on how the virus affects the inner ear was gradually increased. To determine the etiopathogenesis of the SNHL damage, an accurate anamnesis is required in order to establish the onset of SNHL with a clear temporal connection to COVID-19 infection (therefore, having excluded a pre-existent SNHL). Unfortunately, it was not possible to conduct a comprehensive audiological examination of infected and isolated patients or those admitted to the intensive care unit (ICU). In addition, in certain cases, the identification of SNHL was often delayed, and therefore it was difficult to establish a diagnosis and prompt and adequate treatment [3].

The aim of this paper is to evaluate the possible association between SNHL and COVID-19 infection through a systematic literature review.

## 2. Materials and Methods

The authors performed a literature search of English-language studies focusing on a new SNHL onset of COVID-19 patients via the online database MEDLINE.

The keywords ((“COVID-19” [all fields]) AND “Hearing Loss” [all fields]) OR ((“COVID-19” [all fields]) AND “inner ear” [all fields]) were used to select the studies of interest.

The search covered papers published from 1 January 2020; the final literature search was completed on 15 March 2022.

Inclusion criteria:

- Established SARS-CoV-2 infection (proved by PCR on rhino-pharyngeal swab or serology).
- Cases of new onset SNHL evaluated by pure tone audiometry.
- Temporal correlation between the two events.
- Exclusion criteria:
- Conductive or mixed hearing loss.
- Non-confirmed SARS-CoV-2 infection
- Unclear temporal link between the two events.
- Studies published not in the English language.
- Studies with duplicated data.

The search indicated a total of 254 candidate papers, but only 20 papers described SNHL as being directly related to or associated with the SARS-CoV-2 infection.

**Figure 1:**
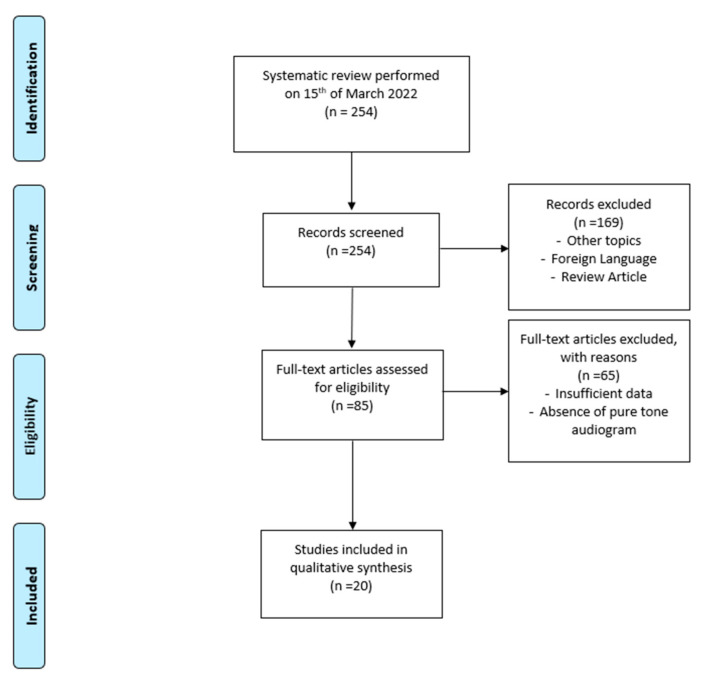
Literature evaluation and selection, according to PRISMA criteria (http://www.prisma-statement.org/ (accessed on 1 April 2022).

The review was performed according to the Preferred Reporting Items for Systematic Reviews and Meta-Analysis (PRISMA) guidelines; the flow diagram is illustrated in Figure 1.

## 3. Results

The literature search identified 20 papers, among case reports and case series, accounting for a total of 63 patients affected by COVID-19 who reported a new SNHL onset (see Table 1).

Four papers were case studies, while sixteen were single-case reports. The year of publication ranged from 2020 to late 2021. The reported mean patient age was 43.4 years (range: 18–72 years old) and the male-to-female-patient ratio was = 1.42.

All patients underwent pure tone audiometry, which disclosed a unilateral SNHL in 36 patients and a bilateral in 27.

**Figure 2:**
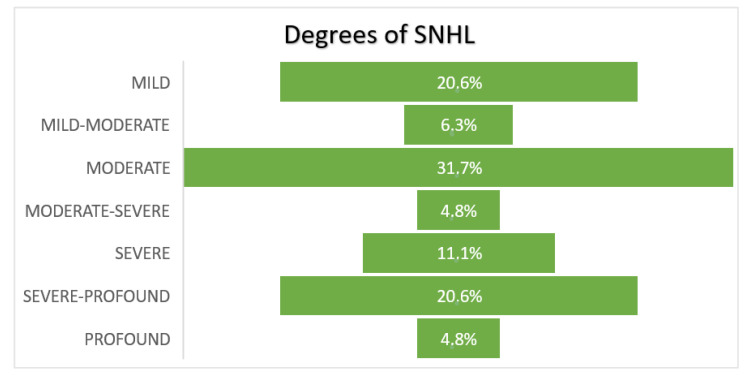
Severity of hearing loss in the patients included in the study.

The degree of hearing loss ranged from mild to moderate in 58.7% of the patients, from moderate to severe in 4.8%, and from severe to profound in 36.5% (see Figure 2).

**Figure 3:**
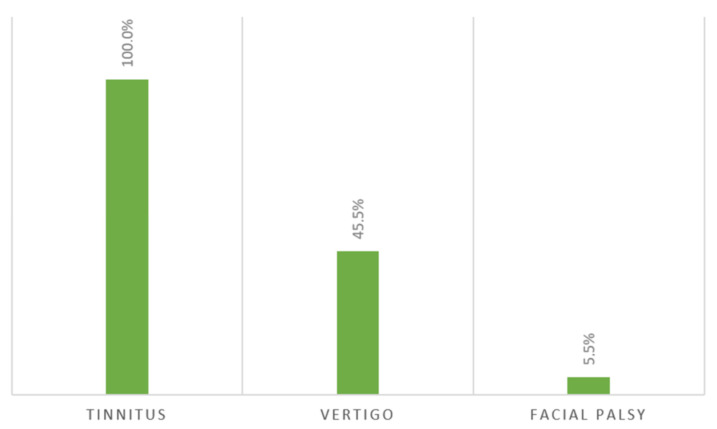
Reported symptoms associated with non-isolated SNHL in COVID-19 patients.

In 12.7% of the patients, SNHL was described as an isolated symptom; whilst, in 87.3%, it was associated with other symptoms (see Figure 3). Interestingly, in three cases, facial palsy incidents were associated with SNHL.

In 25% of the SNHL patients, a chemosensory dysfunction (anosmia, hyposmia, ageusia, and dysgeusia) was also described.

Most of the patients (84.1%) underwent magnetic resonance imaging (MRI), which presented abnormal findings in 11.5% of them (see the paragraphs below). All patients with abnormal MRIs had a severe to profound SNHL, bilateral in 50% of the cases and accompanied by other manifestations, such as tinnitus, vertigo, or facial palsy. None of these patients reported a full recovery of hearing.

The radiological alterations were considerably different:

- Pronounced contrast enhancement of the cochlea (Dagen, 5) or of the VIII and VII nerves (Ozer, 15 and Jeong, 19);
- Hemorrhagic lesion of cerebral parenchyma (Lamounier, 9);
- Intra-labyrinth micro-hemorrhages (Chern, 11);
- Signs peculiar of a specific disease, such as Susac syndrome (Raymaekers, 23).

Otoacoustic Emission (OAE) testing resulted as “REFER” in 75% of tested cases, suggesting probable outer-hair cell damage. Acufenometry, performed only on five patients, identified a high-pitched tinnitus in four subjects and a lower-pitched tinnitus in one.

**Figure 4:**
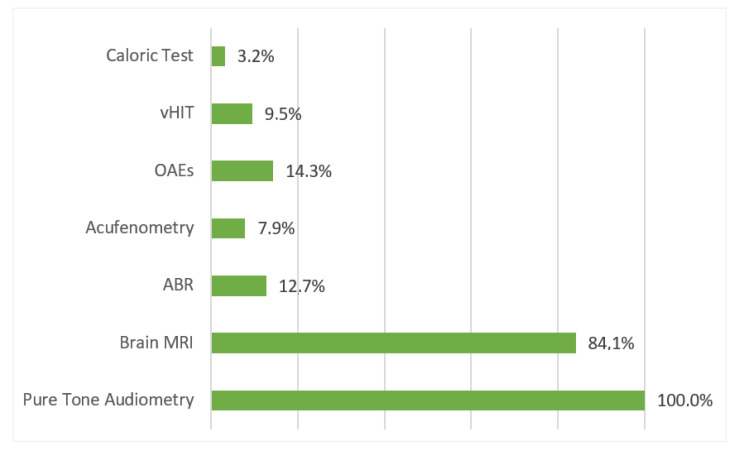
The frequency of audiological diagnostic tests that were used to assess the hearing status of the COVID-19 patients.

Among the patients complaining of vertigo, 24% underwent a video head impulse test (vHIT), which disclosed normal findings in all, except in 2 cases, while only two patients were assessed with videonystagmography (VNG) with a caloric test. A summary of the clinical protocols used to assess the patients is reported in Figure 4.

Two patients presented several associated symptoms, and additional investigations were considered necessary in order to identify a particular underlying pathology. Electromyography and lumbar puncture were required to diagnose Guillain–Barrè syndrome and establish appropriate treatment in a pregnant woman with rapid bilateral facial weakness, lower extremity paresthesia, and audiovestibular deficit, including SNHL. In a patient with sudden SNHL, associated with disequilibrium and confusion, the brain MRI and the subsequent fluorescein angiography identified Susac syndrome, a rare disease characterized by encephalopathy, SNHL, and branch retinal artery occlusions. The possible role played by SARS-CoV-2 in these peculiar cases is still under discussion.

Furthermore, patients affected by COVID-19 were also treated with ototoxic drugs. The administration of potentially ototoxic medications, such as hydroxychloroquine, furosemide, and azithromycin, and antiviral drugs, such as remdesivir and favipiravir, was reported in 12 out of 63 cases. However, there was no clear link between hearing loss and these drugs, which, in some cases, were administered after the onset of audiological symptoms.

Specific therapy for SNHL was described in 86% of subjects. Their treatment included steroids administered orally, intravenously, or/and by intratympanic injection; other reported treatments include hyperbaric oxygen therapy, mesoglican, monoclonal antibodies, and immunosuppressive therapy (as seen in Guillain–Barré and Susac syndrome cases).

A wide-range report of the outcomes was not provided in all studies, especially in the early ones. A full recovery was reported for only 12.5% of patients, while a hearing threshold improvement (from mild to almost complete) was reported for 35.9% of the cases. Three patients underwent cochlear implantation for hearing rehabilitation; in one case, the surgery was performed rapidly, after the cerebral MRI showed multiple cochlear fibrotic loci.

## 4. Discussion

Sensorineural hearing loss (SNHL), tinnitus, and/or vertigo have been described to occur during and following COVID-19 infection. To date, different hypotheses have been proposed to explain the etiopathogenesis of neurological symptoms reported during the acute and post-acute phases of the infection. It is likely that many factors, or a combination of mechanisms, may be involved in the etiopathogenesis of different symptoms, including SNHL. These could consist of hypoxia, immune-mediated damage, coagulative disorders, and viral direct invasion/damage [24].

Since the inner ear is reported to be vulnerable to viruses, direct damage can be hypothesized. The high rate of chemosensory impairment in COVID-19 patients endorses the neuro-invasiveness features of SARS-CoV-2, and the olfactory nerve may represent the virus entry point to the central nervous system. In fact, 25% of patients also included in the present review reported taste and smell dysfunction, while facial nerve disorder was reported in only 4.6% of cases. Moreover, a direct involvement of the nervous system was revealed by the brain MRI (in two cases), which showed pronounced contrast enhancement of the VIII and VII nerves [15,19].

A mechanism, proposed for explaining the direct damage related to SARS-CoV2 infection, could involve the receptor of the angiotensin-converting enzyme 2 (ACE 2). It is the binding partner for SARS-CoV2 in human cells, necessary for the interaction with the viral spike proteins. Since the spread of the pandemic, various authors have identified ACE 2 receptors in tissues other than the respiratory tract, where their presence is well known, in order to explain COVID-19 extrapulmonary symptoms [25]. ACE2 receptors have been found in the Eustachian tube, middle ear, and cochlea (hair cells) of both animal models and humans, suggesting that these tissues are susceptible to a SARS-CoV-2 infection [19,26].

The hypothesis of possible direct hearing damage has been explored through the audiological evaluation of newborns intrauterinely exposed to SARS-CoV-2. Nevertheless, a clear link between congenital infection and an increased risk of hearing loss has not been established [27,28,29,30].

The vertical transmission of COVID-19 has been reported to be very low and, to date, only one study has shown an increased incidence of hearing loss associated with SARS-CoV-2 positivity during pregnancy [31]; however, maternal infection appears to be related to reduced fetal growth and increased perinatal mortality [32].

Furthermore, since the cochlear arterial supply is terminal, with several intra- and inter-individual vascularization variants, a microvascular disorder linked to the infection/inflammation might cause sudden unilateral/asymmetrical hearing loss (indirect ischemic damage). Additionally, micro-hemorrhages of the cerebral parenchyma [9] and labyrinth [11] were revealed by brain MRIs for two patients who experienced hearing loss (indirect hemorrhagic damage). Regarding micro-vascular damage, the post-mortem histopathological brainstem examination of COVID-19 patients revealed a degeneration of the basal lamina of endothelial cells and a congestion of blood vessels, as well as perivascular inflammatory infiltration, constituted by macrophages, astrocytes, and lymphocytes [33].

Several authors have reported that SARS-CoV-2 could stimulate the production of proinflammatory cytokines and promote the onset of autoimmune response; this could potentially play a role in the pathogenesis of Susac syndrome and Guillain–Barré syndrome. This association, however, is still debated.

A corrected differential diagnosis is required when approaching patients with hearing loss, possibly related to COVID-19. It is crucial to establish a clear temporal link between SNHL onset and a confirmed SARS-CoV-2 infection (at the PCR for the rhino-pharyngeal swab), similar to the reports included in this review.

Very few studies have reported a decreased overall incidence of sudden SSNHL during the COVID-19 pandemic [34,35], possibly due to the widespread use of medical masks in association with social distancing. These aspects may have limited the spread of other viral infections and, possibly, also the onset of other SSNHL cases. Furthermore, it has been reported that, during the lockdown periods, patients may have delayed or avoided medical care, especially for non-life-threating conditions, and therefore SSNHL cases may have been underestimated [34,35].

Unfortunately, since it is not possible to conduct a comprehensive audiological examination of infected and isolated patients, or of those admitted to the intensive care unit (ICU), the diagnosis of SNHL has often been delayed, and this can potentially delay the onset of proper treatment, as in some of the reported cases, with effects on outcomes: the SNHL full-recovery rate in the present review is only 12.5%.

The development of prevention strategies, based on the reports available to date, is difficult. According to the available literature, in rare cases, COVID-19 may be associated with the onset of severe hearing loss. Particularly in these cases, a prompt diagnosis and a prompt therapeutic intervention is crucial.

Finally, since December 2020, as the European Medicines Agency (EMA) approved the first vaccine against COVID-19 (Pfizer-BioNTech), a large immunization campaign commenced and potential vaccine-related adverse effects, including audio-vestibular effects, were reported. Few case reports and small sample studies suggested a possible link between vaccination and an increased risk of SNHL, however with a very low level of evidence [36,37,38,39,40,41,42]. Even if a clear and defined relationship between the COVID-19 vaccination and SSNH cannot be identified in the literature to date, we expect that further studies and reports on this topic could be available in the future.

## 5. Conclusions

Currently available data show that SARS-CoV-2 may hamper cochlear function. However, since the available reports are limited and often anecdotal, further studies are necessary, particularly to evaluate the possible etiopathogenetic features between SARS-CoV-2 and SSNHL.

Nonetheless, it is likely that SNHL could be included among the manifestations of the so-called “long COVID” syndrome.

## Figures and Tables

**Table 1:** List of papers reporting a new SNHL onset, post COVID-19 infection (Ref. = number of bibliographic references, # = number of patients).

Authors	Ref.	Year	Country	#	Age (Years)	Sex
**Kilic O. et al.**	[4]	June 2020	Turkey	1	29	M
**Degen C. et al.**	[5]	June 2020	USA	1	60	M
**Abdel Rhman S. et al.**	[6]	July 2020	Egypt	1	52	M
**Lang B. et al.**	[7]	October 2020	Ireland	1	30	F
**Koumpa FS. et al.**	[8]	October 2020	UK	1	45	M
**Lamounier P. et al.**	[9]	November 2020	Brazil	1	67	F
**Karimi-Galougahi M. et al.**	[10]	December 2020	Iran	3	22	M
					40	F
					23	F
**Chern A. et al.**	[11]	January 2021	USA	1	18	F
**Aasfara J. et al.**	[12]	January 2021	Morocco	1	36	F
**Beckers E. et al.**	[13]	March 2021	Belgium	1	53	M
**Edwards M. et al.**	[14]	June 2021	UK	1	68	F
**Ozer F. et al.**	[15]	July 2021	Turkey	1	62	F
**Ricciardiello F. et al.**	[16]	July 2021	Italy	5	26	F
					22	M
					61	M
					30	M
					46	F
**Gerstacker K. et al.**	[17]	August 2021	Germany	1	38	M
**Shah S.M. et al.**	[18]	August 2021	UK	4	46	F
					43	F
					54	F
					51	M
**Jeong M. et al.**	[19]	October 2021	USA	10	Mean 48.8	6 M
					(range 22–72)	4 F
**Pokharel S. et al.**	[20]	October 2021	Nepal	1	27	M
**Yaseen N.K. et al.**	[21]	October 2021	Iraq	26	Mean 39.23	6 M
					(range 21–66)	21 F
**Asfour L. et al.**	[22]	November 2021	USA	1	34	M
**Raymaekers V. et al.**	[23]	November 2021	Belgium	1	40	M
